# CCL17/TARC and CCR4 expression in Merkel cell carcinoma

**DOI:** 10.18632/oncotarget.25836

**Published:** 2018-07-31

**Authors:** Kashif Rasheed, Ibrahim Abdulsalam, Silje Fismen, Øystein Grimstad, Baldur Sveinbjørnsson, Ugo Moens

**Affiliations:** ^1^ Molecular Inflammation Research Group, Department of Medical Biology, Faculty of Health Sciences, University of Tromsø, N-9037, Tromsø, Norway; ^2^ Department of Pathology, University Hospital of Northern Norway, N-9038, Tromsø, Norway; ^3^ Department of Dermatology, University Hospital of Northern Norway, N-9038, Tromsø, Norway

**Keywords:** Merkel cell carcinoma, inflammation, cytokines, CCL17/TARC, CCR4

## Abstract

Merkel cell carcinoma (MCC) is a rare, highly aggressive neuroendocrine skin cancer. In more than 80% of the cases, Merkel cell polyomavirus (MCPyV) is a causal factor. The oncogenic potential of MCPyV is mediated through its viral oncoproteins, large T antigen (LT) and small t antigen (sT). To investigate the role of cytokines in MCC, a PCR array analysis for genes encoding inflammatory cytokines and receptors was performed on MCPyV-negative and MCPyV-positive MCC cell lines, respectively. We detected an increased expression of CCL17/TARC in the MCPyV-positive MKL2 cell line compared to the MCPyV-negative MCC13 cell line. Transfection studies in MCC13 cells with LT expression plasmid, and a luciferase reporter plasmid containing the CCL17/TARC promoter, exhibited stimulated promoter activity. Interestingly, the ectopic expression of CCL17/TARC upregulated MCPyV early and late promoter activities in MCC13 cells. Furthermore, recombinant CCL17/TARC activated both the mitogen-activated protein kinase and the NF-κB pathways. Finally, immunohistochemical staining on human MCC tissues showed a strong staining of CCL17/TARC and its receptor CCR4 in both LT-positive and -negative MCC. Taken together, CCL17/TARC and CCR4 may be a potential target in MCC therapy providing MCC patients with a better overall survival outcome.

## INTRODUCTION

Merkel cell carcinoma (MCC) is a rare, highly aggressive neuroendocrine skin cancer [1, 2]. In 2008, using digital transcriptome subtraction, a new virus belonging to the family of Polyomaviruses, and hence named Merkel cell polyomavirus (MCPyV), was identified in MCC. Worldwide studies have shown that approximately 80% of all examined MCC contain clonal integrated MCPyV DNA [3], thus indicating that MCPyV is associated with the etiology of MCC [4, 5]. MCPyV has a circular, double-stranded DNA genome of approximately 5.4 kb [6]. The viral genome possesses the typical polyomavirus tripartite organization, with an early region encoding the regulatory proteins large T (LT) antigen and small T (sT) antigen [7], the late region encoding the capsid proteins [8], and a non-coding control region encompassing the origin of replication and transcription regulatory elements [9]. In addition, the early region encodes the 57kT antigen and a protein called alternative LT ORF (ALTO), although their function remains unknown [7]. All MCPyV-positive (MCPyV+) MCC express a C-terminal truncated form of LT antigen that has lost its DNA binding and p53 interaction domains, but retains the ability to interact with retinoblastoma protein pRb [10]. Both sT and full-length LT, as well as truncated LT, have been shown to possess oncogenic potential in both cell culture and animal models [11-14]. The proliferation of MCC cell lines depends on the expression of LT [15], while the role of sT has been disputed [16, 17].

Inflammation has long been associated with tumor progression [18]. Many cancers arise from sites of infection, chronic irritation and inflammation, and inflammatory signaling pathways are often activated by oncogenic mutations [19]. Chemokines are a family of cytokines that regulate leukocyte trafficking in immunity and inflammation, playing a significant role in processes attributed to tumorigenesis, such as tumor cell survival, senescence, angiogenesis, metastasis and immune escape. The aberrant expression of chemokines and chemokine receptors in tumors may regulate the trafficking of leukocytes into the tumor microenvironment [20]. Chemokines secreted within the tumor microenvironment may also act in an autocrine manner to promote the proliferation and migration of the tumor cells. Chemokine (C-C motif) ligand 17/thymus and activation-regulated (CCL17/TARC) is a member of the CC chemokine family, and is highly expressed by thymus and other cells, including keratinocytes, endothelial cells, dendritic cells, bronchial epithelial cells and fibroblasts [21]. CCL17/TARC acts as a chemoattractant, which primarily aids the recruitment of CD4^+^ T regulatory cells and Th2, in addition to Th17 cells [22, 23]. The effect of CCL17/TARC is mediated by the chemokine receptor CCR4 [24, 25].

In this study we compared the cytokine expression pattern in MCPyV-positive with MCPyV-negative MCC cells and examined the role of the viral protein LT on cytokine expression. In addition, we investigated whether cytokines have an impact on viral expression.

## RESULTS

### Differential expression profile of inflammatory modulators in MCPyV-negative and MCPyV-positive MCC cell lines

**Figure 1:**
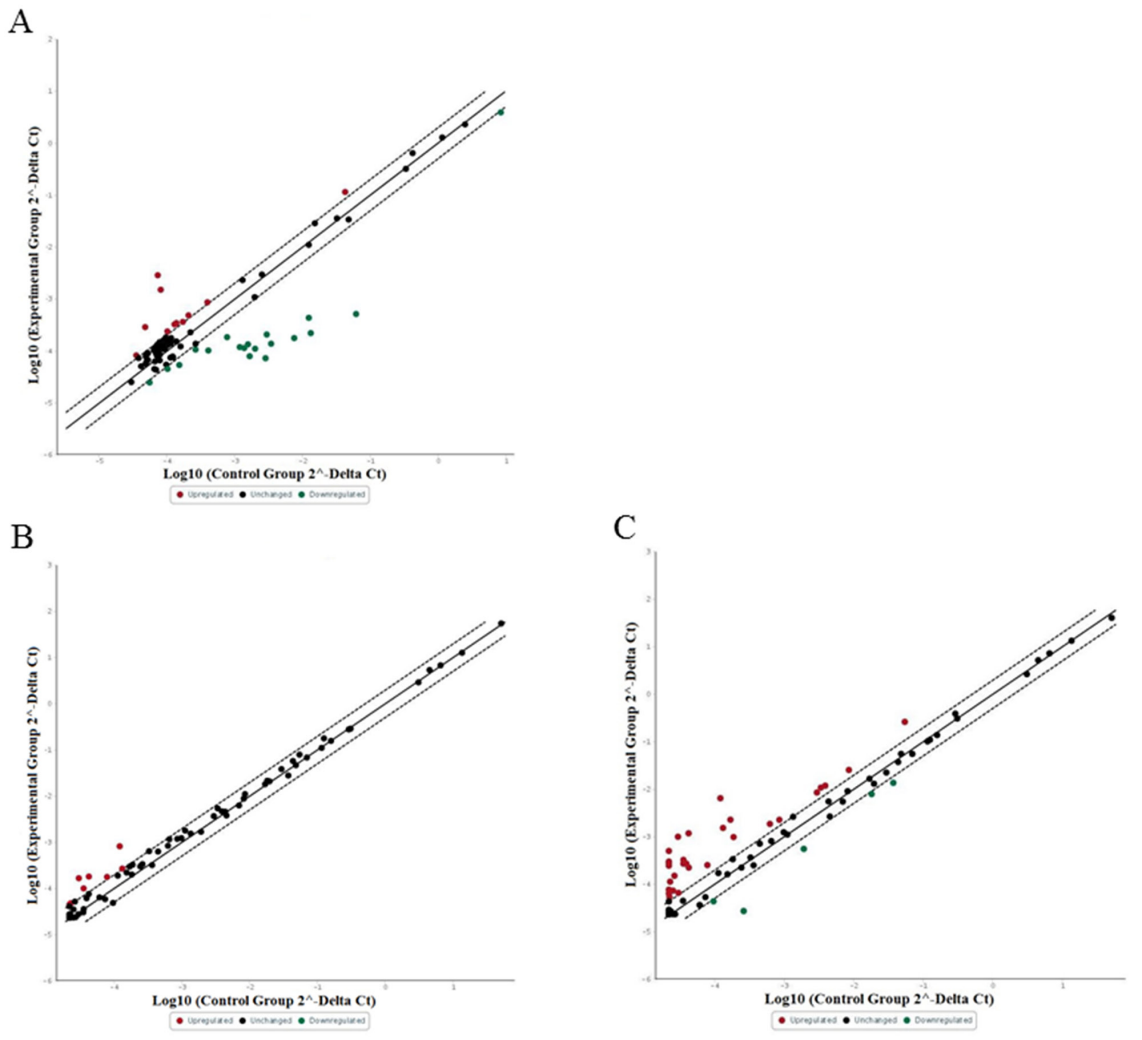
Relative expression comparison of 84 inflammatory cytokines and receptors genes between MCPyV-associated and non-associated Merkel cell carcinoma The figures depict a log transformation plot of the relative expression level of each gene (2^-ΔCt^) between **(A)** MCC13 cells vs. MKL-2 cells, **(B)** full-length LT vs. empty vector transfection in MCC13 cell line and **(C)** MKL-2 truncated LT vs. empty vector transfection in MCC13 cell line. The dotted lines indicate a two-fold change in gene expression threshold.

Inflammatory mediators such as cytokines are known to play a role in cancer. This prompted us to compare the expression of 84 inflammatory cytokines and cytokine receptors in a MCPyV-negative (MCC13) and a MCPyV-positive MCC cell line (MKL-2), respectively. All 84 human inflammatory cytokines and receptor transcripts were detectable (Cq < 35) by RT profiler PCR array. Out of 84 transcripts, with the fold changes critical value set ≥ 2 fold differentiated, the expression of 11 (13.09%) were upregulated and the expression of 18 (21.42%) were downregulated in MKL-2 cells compared with MCC13 cells, while 55 (65.47%) of the genes had comparable transcription levels in both cell lines (Figure 1A) (data not shown).

### Expression analysis of cytokines and their receptor in MCPyV-negative MCC13 cells and MCC13 cells transiently expressing exogenous LT

Because the LT of polyomaviruses is known to affect viral and cellular gene expression (30), we wanted to examine whether LT may be responsible for the differential expression of cytokines and their receptors in these MCC cell lines [10, 26]. Therefore, the eukaryotic expression vectors for MKL-2 LT, but also for the LT of two other virus-positive MCC cell lines (MKL-1 and MS-1), were generated. These virus-positive expression plasmids containing full-length LT and truncated LT (MKL-1, MKL-2 and MS-1) were then confirmed by sequencing (Supplementary Figure 1). A Western blot of lysates from MCC13 cells transfected with these expression plasmids confirmed the presence of LT with a correct predicted molecular mass (Supplementary Figure 2). Next, we transfected MCC13 cells with pcDNA3-full-length LT, pcDNA3-MKL-2, or empty vector pcDNA3 as a control, and measured the transcript levels of the 84 inflammatory cytokines and receptors by qPCR. For cells transfected with full-length LT expression plasmid, 71 out of 84 human inflammatory cytokines and receptor transcripts were detectable (Cq < 35), whereas the transcript for the remaining 13 genes was undetectable or had Cq values ≥ 35 by RT profiler PCR array. Out of 84 transcripts, with the fold changes critical value set ≥ 2, 8 (9.52%) genes were upregulated, while 76 (90.48%) genes did not show any effect or undetectable compared with empty vector transfected cells (Figure 1B) (data not shown). For cells transfected with MKL-2 truncated LT expression plasmid, 70 out of 84 human inflammatory cytokines and receptor transcripts were detectable (Cq < 35), while of the remaining, 14 were undetectable or had Cq values ≥ 35 by RT profiler PCR array. Out of 84 transcripts, with the fold changes critical value set ≥ 2, 32 (38.09%) genes were upregulated and 4 (4.76%) genes were downregulated, while 48 (57.14%) genes did not show any effect or undetectable compared with the control cells (data not shown) compared to MCC13 cells transfected with an empty vector (Figure 1C). One of the genes whose transcript levels were consistently upregulated in MKL-2 cells, and in MCC13 cells transiently expressing full-length or truncated MKL-2 LT compared to MCC13 cells, was CCL17/TARC.

### MCPyV large T antigen induces CCL17/TARC promoter activity

**Figure 2:**
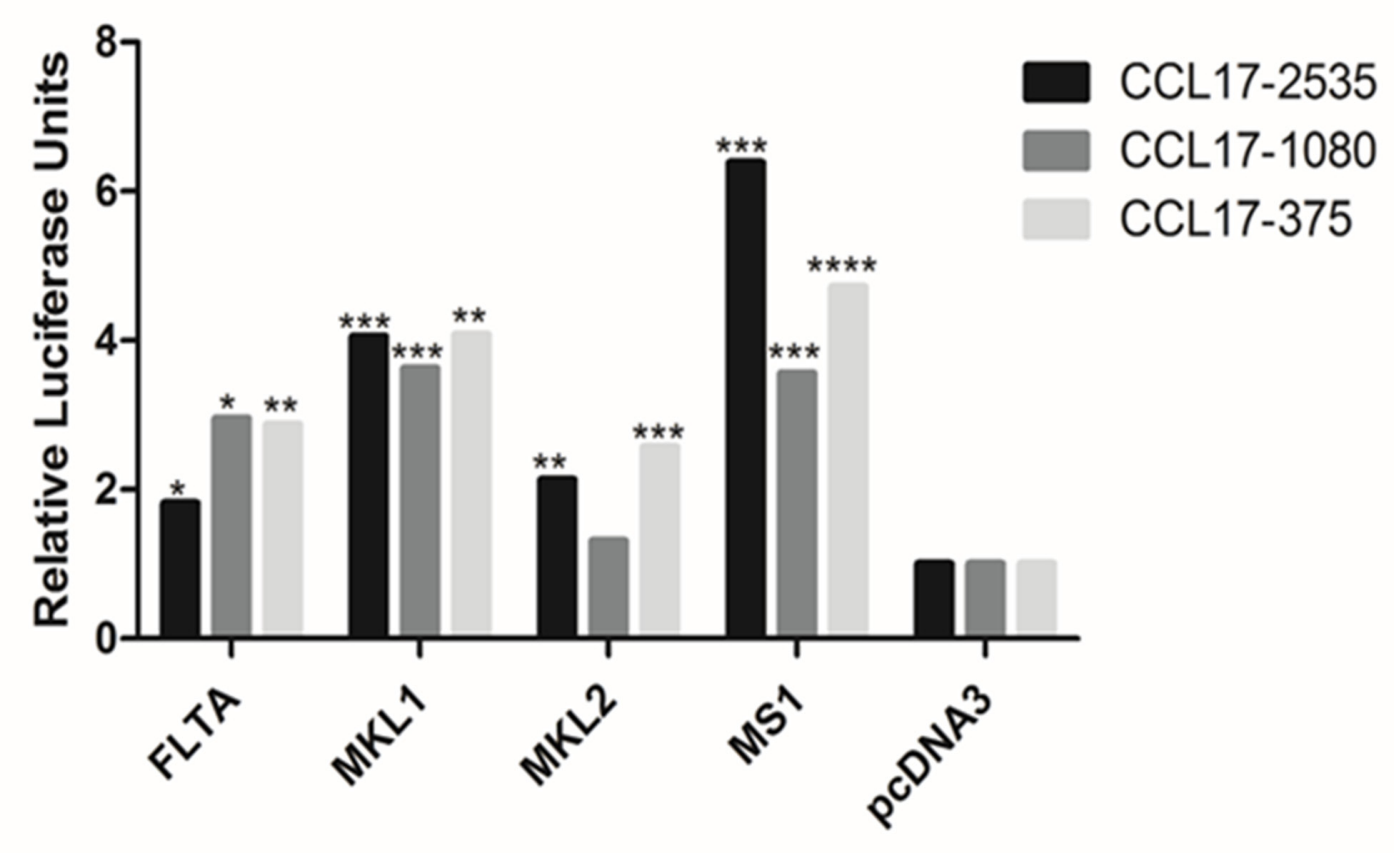
Effect of full-length and truncated (MKL-1, MKL-2 and MS-1) LT on CCL17/TARC promoter activity in MCC13 cells Cells were transfected with a luciferase reporter vector driven by CCL17/TARC promoter fragments spanning nucleotides -2535/+ 40, -1084/+ 40 or -375/+40, respectively. Expression plasmid for full-length LT (FLTA), MKL-1, MKL-2 or MS-1 LT, or pcDNA3 control, were co-transfected. Luciferase activity was assessed after an overnight cultivation of transfected cells. The promoter activity in presence of pcDNA3 was arbitrary set as 1.0 and the promoter activity measured in the presence of LT was related to this. Each bar represents the average of three independent parallels +SD. The experiment was repeated two more times, and similar results were obtained. Luciferase values were normalized with a total protein in each sample. P^*^ ≤ 0.05, P^**^ ≤ 0.01, P^***^ ≤ 0.001 and P^****^ ≤ 0.0001.

Because CCL17/TARC expression was shown to be upregulated in cells that were either MCPyV-positive or that expressed a full-length or truncated LT, we tested whether MCPyV LT could induce CCL17/TARC promoter activity. For this purpose, MCC13 cells were transiently co-transfected with a plasmid encoding a full-length or truncated LT (MKL-1, MKL-2 and MS-1), and with a luciferase reporter driven by different fragments of the CCL17/TARC promoter (fragment -2535/+ 40, -1084/+ 40 or -378/+40, respectively). The empty vector pcDNA3 was used as a control. Full-length, as well as the truncated LT versions MKL-1 and MS-1 significantly stimulated the CCL17/TARC promoter activity. The largest CCL17/TARC promoter fragment (-2535/+40) was more potently activated than the shorter promoter fragments (-1084/+40 and -375/+40, respectively) by LT MKL-1 and MS-1, while comparable full-length LT-mediated transactivation the three different CCL17/TARC promoter fragments. MKL-2 LT significantly stimulated the activity of the CCL17/TARC promoter encompassing nucleotides -2535/+40 and -375/+40, but induced only slightly, but statistically insignificant the CCL17/TARC -1080/+40 promoter sequence (Figure 2). These results confirm the qPCR data showing that CCL17/TARC expression is higher in LT expressing MCC13 compared to MCC13 cells. The enhanced CCL17/TARC transcript levels in MKL-2 cells, compared to the virus-negative MCC13 cells, may at least be partially triggered by LT. In contrast, sT did not stimulate the activity of any of the CCL17 promoter fragments or increase sT protein levels when expressed in MCC13 cells (results not shown).

### CCL17/TARC ectopic effect on MCPyV early and promoter activity

**Figure 3:**
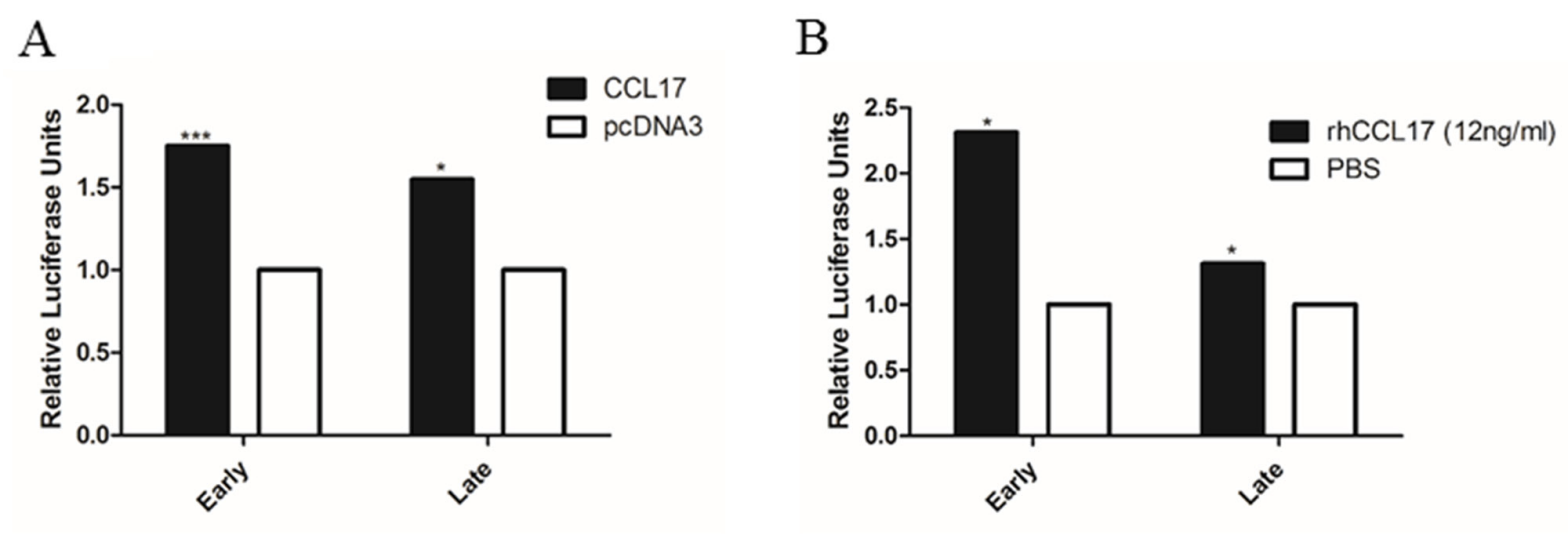
CCL17/TARC stimulates MCPyV early and late promoter activity **(A)** MCC13 cells were seeded, and after 24 hrs the cells were serum starved. After serum starvation for 24 hrs, the cells were co-transfected with either CCL17/TARC expression plasmid or empty expression vector pcDNA3.1, and luciferase reporter plasmid containing either the MCPyV early or the late promoter. Luciferase activity was measured 24 hrs after transfection. The activity of the MCPyV early (respectively late) promoter in the presence of pcDNA3 was arbitrary set as 1.0 and the activity in the presence of CCL17 was related to this. **(B)** Cells were transfected as in (A), and were then exposed to rhCCL17/TARC (12ng/ml) or vehicle (PBS) for 4 hrs with a subsequent analysis of luciferase activity. Each bar represents the average of three independent parallels +SD^*^P < 0.05, ^***^P < 0.001.

Cytokines such as IL-1β, TGF β and TNF-α have been shown to stimulate the activity of human polyomavirus promoters [27-30]. Therefore, we investigated whether CCL17/TARC could exert an effect on the MCPyV early and late promoter activity. MCC13 cells were transfected with a luciferase reporter plasmid containing, either the early or late MCPyV promoter, and cells were either co-transfected with a CCL17/TARC expression plasmid or treated with recombinant human CCL17/TARC protein. Both an ectopic expression of CCL17/TARC and administering of recombinant CCL17/TARC to cells resulted in a significant upregulation of both the early and late MCPyV promoter activity (Figure 3).

### Expression of CCL17/TARC and CCR4 in MCC cells

**Figure 4:**
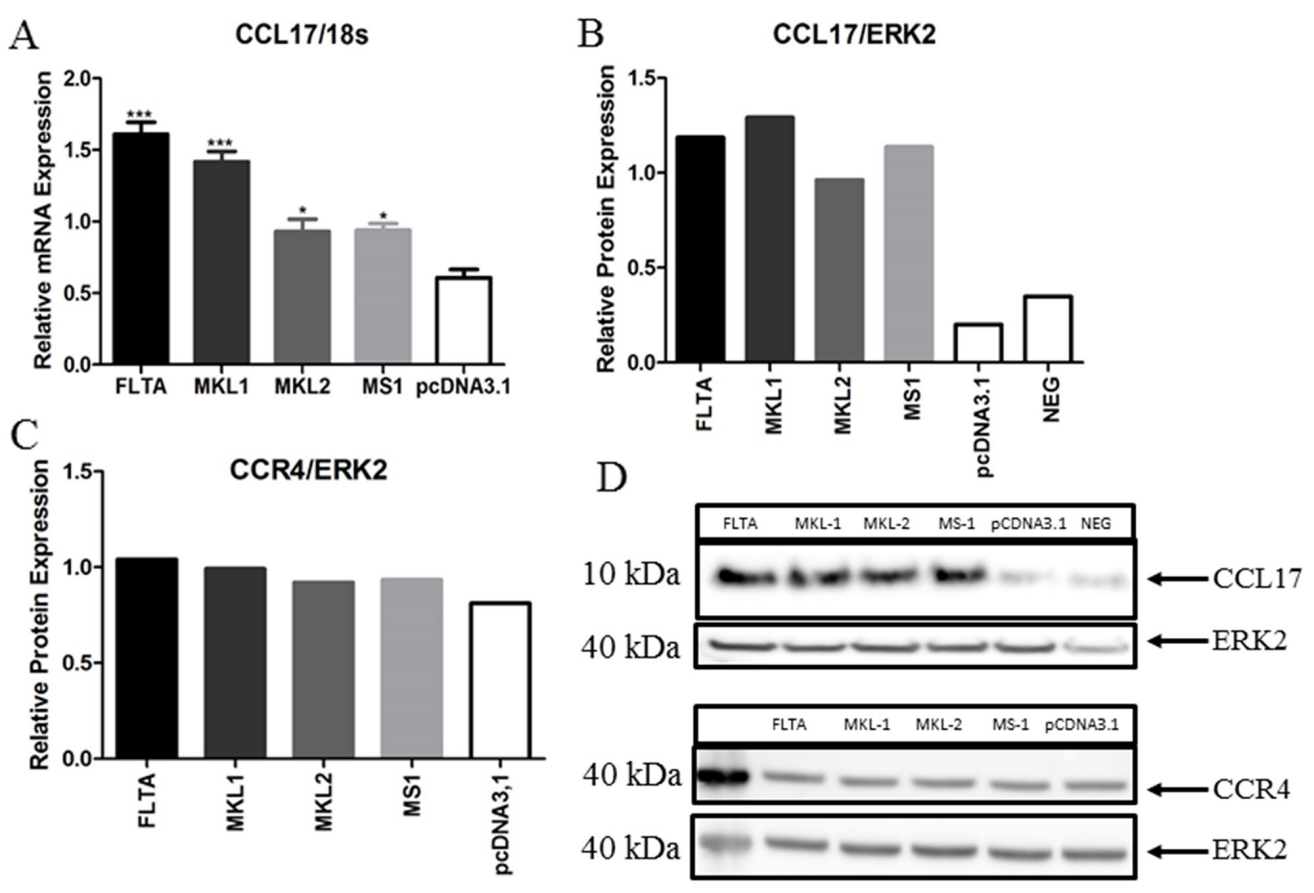
Transient expression of full-length or truncated MCPyV LT increases the transcript and protein levels of CCL17/TARC MCC13 cells were transfected with an empty vector or expression plasmid for MCPyV full-length LT (FLTA), or truncated MKL-1, MKL-2, or MS-1 LT. **(A)** qRT-PCR analysis shows CCL17/TARC mRNA levels normalized with eukaryotic 18S rRNA levels. **(B)** CCL17/TARC protein levels analyzed by Western blotting. The uttermost right lane represents baseline expression of CCL17/TARC by MCC13 cells. **(C)** CCR4 protein levels analyzed by Western blotting. A Western blot with ERK2 antibodies was used as a loading control. **(D)** Representative figures of CCL17/TARC (B) and CCR4 (C) Western blots. The lane most to the left in the lower part contains the protein molecular mass marker (in kDa). Bars in (B) and (C) shows a densitometric scanning of the Western blot signals. P^*^ ≤ 0.05 and P^***^ ≤ 0.001.

To confirm the stimulating effect of full-length and truncated LT on CCL17/TARC promoter activity, we first evaluated the mRNA expression of CCL17/TARC by qPCR in MCC13 cells transfected with expression vector for full-length LT or truncated LT variants. Full-length and MKL-1 LT significantly increased CCL17/TARC expression in the MCC13 cell line (P < 0.01), while MKL-2 and MS-1 LT variants, modestly, but significantly increased CCL17/TARC mRNA levels (Figure 4A). Western blot analysis with anti-CCL17/TARC antibodies confirmed that CCL17/TARC protein levels were increased in MCC13 cells expressing either full-length or truncated LT compared to MCC13 cells (Figure 4B and 4D). In our screening experiments, we also found a slight upregulation of CCR4 mRNA by exogenous expression of both pcDNA3-FLTA and pcDNA3-MKL-2 plasmids. So, to check at the protein level, we conducted a Western blot. We did not find expression at a significant level, but only a slight upregulation of CCR4 in MCC13 cells with an exogenous expression of MCPyV LT (Figure 4C and 4D).

### CCL17/TARC activates the mitogen-activated protein kinase (MAP kinase) and NF-κB pathways in MCC cells

**Figure 5:**
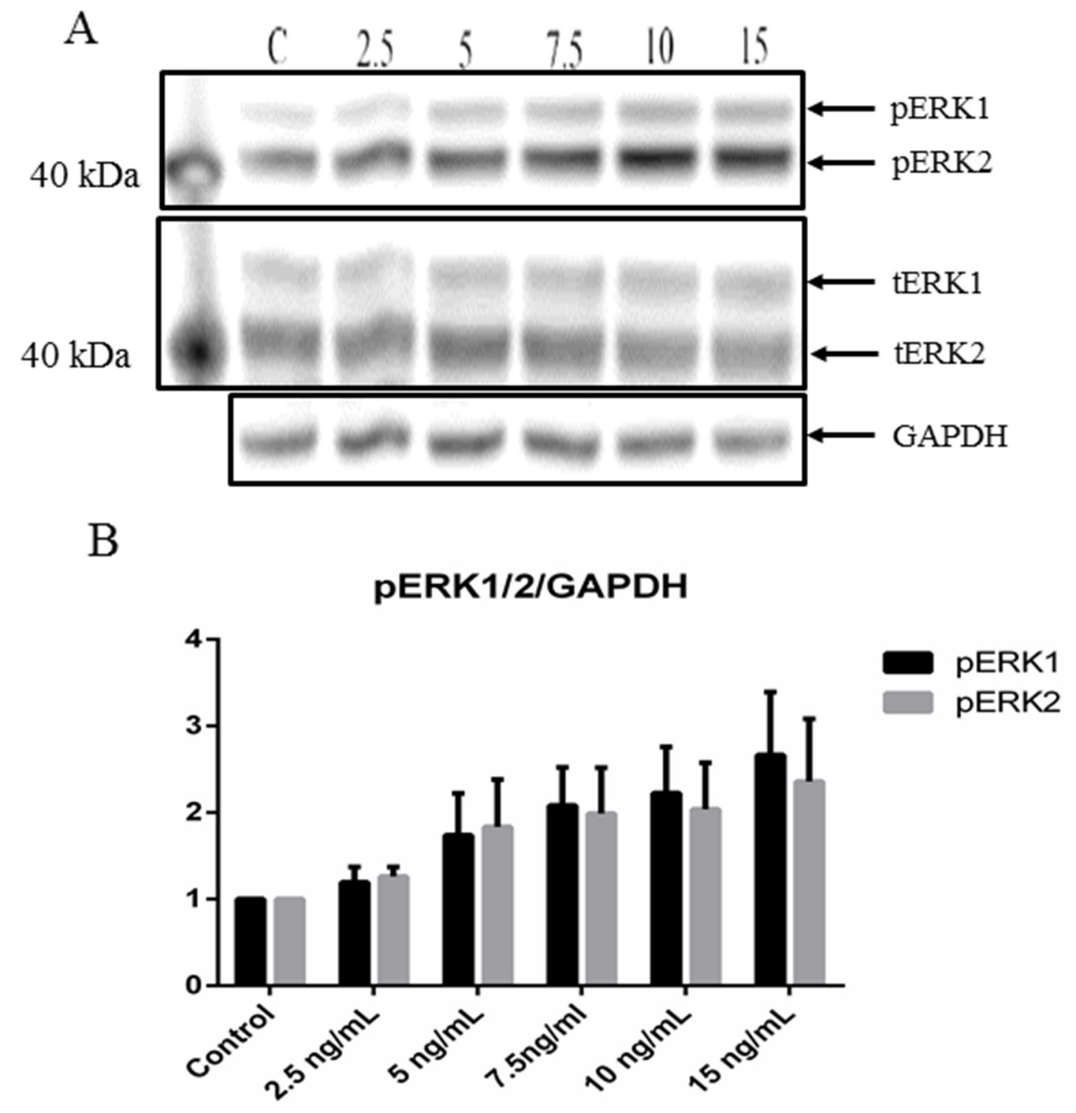
CCL17/TARC upregulates ERK1/2 activity **(A)** PhosphoERK1/2 (pERK1/2) expression levels were determined by Western blot analysis using a phospho-specific antibody detecting Thr202/Tyr204 phosphorylation and compared to total ERK1/2 (tERK1/2) levels using an anti-pan-ERK1/2 antibody. MCC13 (1x10^5^) cells were seeded in a 6-well plate. Cells were then serum-starved for 24 hrs, and thereafter stimulated with either PBS (control or C) or with 2.5, 5, 7.5, 10 and 15 ng/ml of rhCCL17/TARC for 45 min. **(B)** Densitometry scanning represent the expression of pERK1 (respectively pERK2) protein relative to GAPDH.

**Figure 6:**
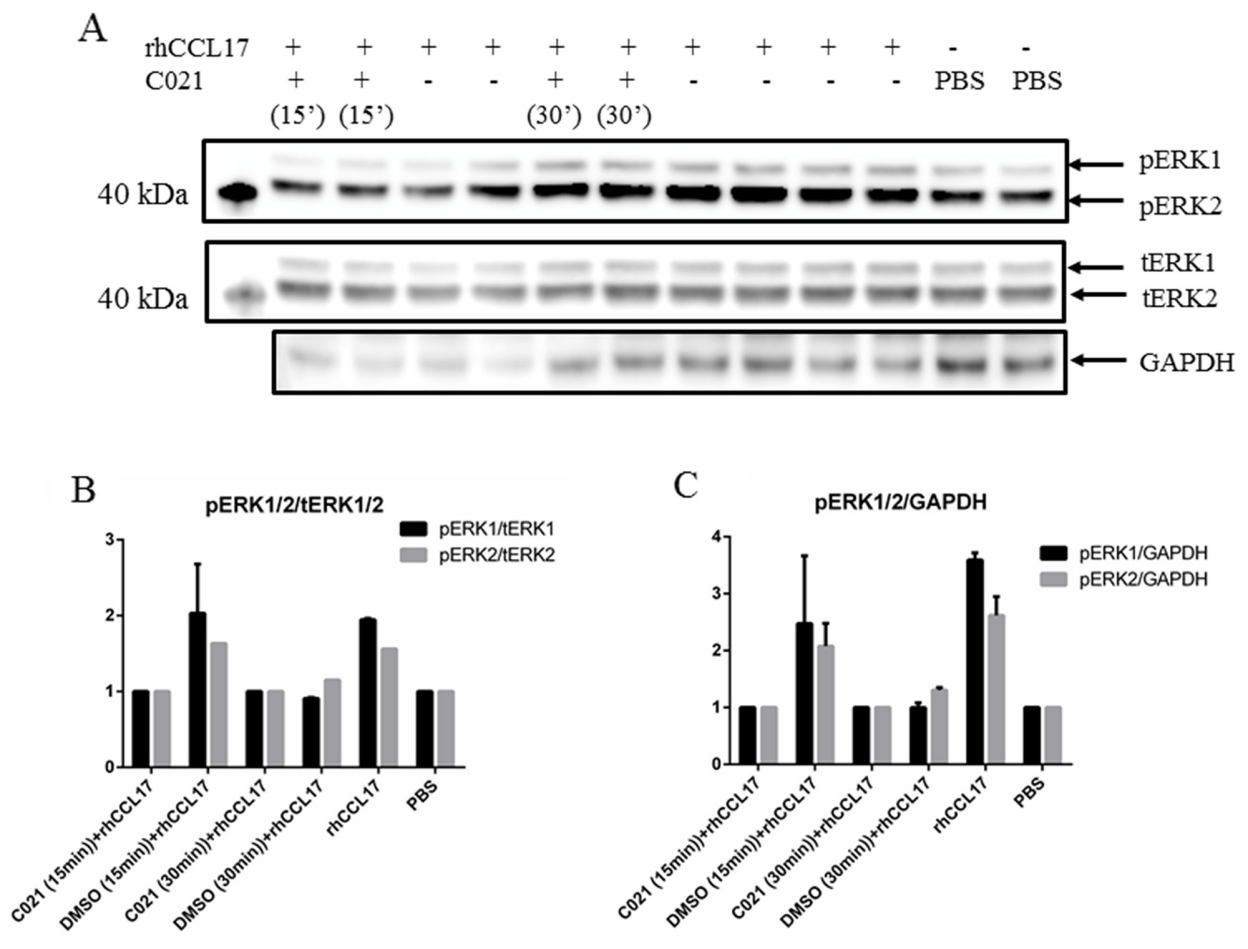
The CCR4 receptor antagonist C021 reduces CCL17/TARC-induced phosphorylation of ERK1/2 **(A)** MCC13 (1x10^5^) cells were seeded in a 6-well plate and were serum starved for 24 hrs. The cells were either pre-exposed to PBS, C021 dihydrochloride (+), a specific CCR4 receptor antagonist, or left untreated (-) for 15 min (15’) or 30 min (30’) as indicated. The cells were subsequently incubated with rhCCL17/TARC (15ng/ml) or left untreated (-) for another 45 min. Cell lysates were prepared and phosphoERK1/2 and total ERK1/2 levels were monitored. GAPDH levels were used as loading control. Densitometry represents the expression of pERK1 (respectively pERK2) protein relative to **(B)** total ERK (tERK1/2) and **(C)** GAPDH.

Both CCL17/TARC and ERK1/2 have been shown to be involved in skin inflammation [31-33], thereby suggesting that CCL17/TARC may activate the MEK1/2-ERK1/2 mitogen-activated protein kinase pathway. To help investigate the effect of CCL17/TARC on MEK1/2-ERK1/2 activation in MCPyV-associated MCC, we stimulated MCC13 cells with recombinant human CCL17/TARC. The cells were stimulated with different concentrations (2.5 ng/ml to 15 ng/ml) and time periods (5 min to 60 min) and we monitored the phosphorylation of ERK1/2 using phospho-specific antibodies and western blotting. Activation of ERK1/2 by rhCCL17/TARC was shown to be concentration dependent (Figure 5). The ERK1/2 phosphorylation activity was inhibited by using a specific CCR4 antagonist (C021 dihydrochloride) (Figure 6).

**Figure 7:**
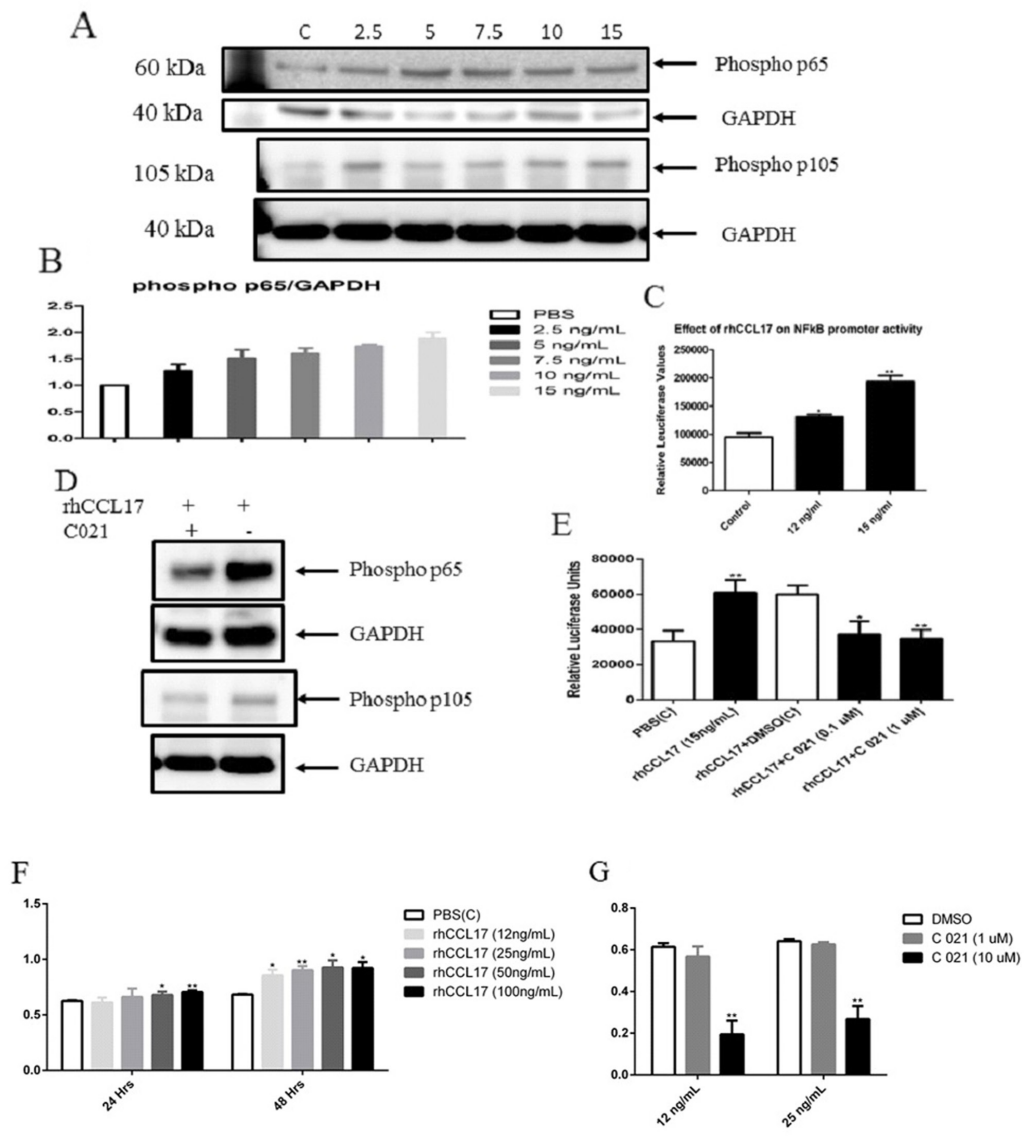
CCL17/TARC upregulated NFκB activity **(A)** NFκB-p65 activation was determined by monitoring phosphorylation of p65 and p105. MCC13 cells (1x10^5^) were seeded in a 6-well plate. Cells were serum starved for 24 hrs, and then stimulated with 2.5, 5, 7.5, 10 and 15 ng/ml of rhCCL17/TARC for 45 min. PBS was used as a control. Relative phospho p65 and phospho p105 were determined by western blot with phosphospecific antibodies. **(B)** Shows densitometry bars of western blot. **(C)** NFκB activity was measured by using a luciferase reporter plasmid containing a NFκB-responsive promoter. Cells were stimulated with 12ng/ml or 15ng/ml of rhCCL17/TARC for 4 hrs. Luciferase values were normalized with total protein. **(D)** The CCR4 receptor antagonist interferes with CCL17/TARC-induced activation of NFκB. Phospop65 and phosphor p105 levels were determined by western blot. MCC13 (1x10^5^) cells were seeded in a 6-well plate and were serum starved for 24 hrs. The cells were pre-incubated with C021 dihydrochloride (0.3μM) for 30 min. The cells were then stimulated with rhCCL17/TARC (15ng/ml) in the presence or without CCR4 receptor antagonist for 45 min. DMSO was used as a control. **(E)** C021 ablates CCL17/TARC-induced activation of an NFκB-responsive promoter. Cells were transfected with the luciferase reporter plasmid with an NFκB responsive promoter and exposed to rhCCL17/TARC (15ng/ml) in the presence or without CCR4 receptor antagonist C012 (0.1 or 1 μM) for 45 min. DMSO was used as a control. Each bar represent the average of three independent parallels. Luciferase values were corrected for protein concentration of the samples. P^*^ ≤ 0.05 and P^**^ ≤ 0.01. **(F)** CCL17/TARC stimulates proliferation of MCC13 cells. Cell were exposed to PBS or increasing concentrations of rhCCL17/TARC /12-100 ng/ml) and cell proliferation was measured after 24 and 48 hrs. Each bar represents the average of three independent parallels. **(G)** Cells were incubated for 45 min with 0.1 or 1 0μM C021 and rhCCL17/TARC (12 or 25 ng/ml) was subsequently added. Proliferation was monitored 24 hrs later. P^*^ ≤ 0.05 and P^**^ ≤ 0.01.

Previous studies have indicated that NF-κB is a target downstream of CCL17/TARC. We found that CCL17/TARC increased the phosphorylation of NF-κB/p65 activity in a concentration-dependent manner (Figure 7A, 7B), which was inhibited by using a specific CCR4 antagonist (Figure 7D, 7E). Furthermore, NF-κB promoter assay confirmed an increased luciferase activity of NF-κB when cells were co-transfected with pCMV2-CCL17 and NF-κB-luc reporter plasmids (Figure 7C).

### CCL17/TARC stimulates cell proliferation of MCC13 cells

Next, we examined the effect of rhCCL17/TARC on MCC13 proliferation. A dose-dependent increase in cell proliferation was observed (Figure 7F). The CCR4 antagonist C021 inhibited CCL17/TARC-induced cell proliferation (Figure 7G).

### CCL17/TARC and its receptor are expressed in MCC tissue samples

**Figure 8:**
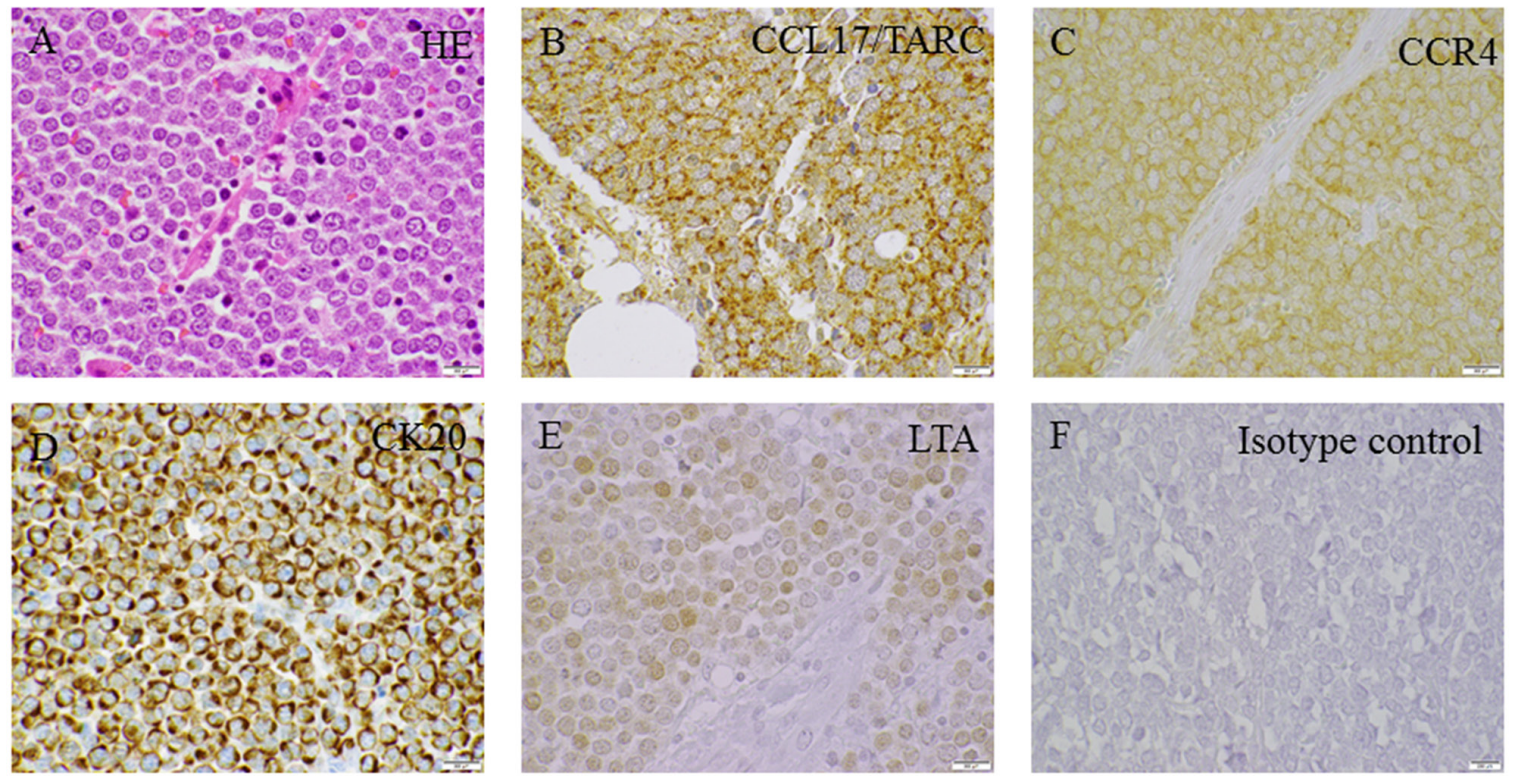
Immunoperoxidase staining of MCPyV-associated MCC primary tumors **(A)** HE, **(B)** CCL17/TARC, **(C)** CCR4, **(D)** CK20, **(E)** LTA and **(F)** Isotype control. The displayed images are representative stainings from a panel of MCC primary tumors (Scale bar= 500 μm).

A total of 23 primary cutaneous MCCs were immunohistochemically stained for LT, CK20, CCL17/TARC and CCR4, respectively. Fifteen out of 23 (65.2%) demonstrated an intranuclear positivity consistent with a positivity for LT. All of the 15 LT-positive tumors demonstrated a uniform positivity for CK20 (dot-like cytoplasmic) and CCL17/TARC (dot-like cytoplasmic). With regard to CCR4, 12 out of 15 (80%) of the LT-positive tumors demonstrated cytoplasmic, membranous positivity for CCR4. Of the remaining eight (34.8%) LT -negative tumors, all of these demonstrated a uniform positivity for CK20, CCL17/TARC and CCR4, respectively (Figure 8).

## DISCUSSION

There is increasing evidence that inflammatory mediators such as chemokines and chemokine receptors are involved in promoting tumor invasion, migration and vascularization [34]. Previous studies have demonstrated the involvement of chemokines, such as CXCL1, CXCL5, CXXC4 and IL20RA in MCPyV-associated MCC [35]. In the current study, we performed the screening of different inflammatory cytokines and receptors, and compared their expression in MCPyV LT-positive and -negative MCC, respectively. Other groups have previously reported that LT and sT have an impact on cytokine expression. Ectopic sT expression in MCC13 cells resulted in decreased IL2, IL-8, CCL20 and CXCL9 expression [36]. Also, expression of full-length LT, truncated LT339, and truncated LT339 plus sT in hTERT-immortalized BJ human foreskin fibroblasts increased the expression of IL-1β, IL-6, IL-8, CXCL1 and CXCL6 [37].

Here we demonstrate that one of the cytokine member whose expression was shown to be significantly enhanced in MCPyV LT-positive cells was CCL17/TARC. CCL17/TARC is a member of CC-motif chemokine family, and is constitutively expressed in thymus and by dendritic cells, endothelial cells, keratinocytes and fibroblasts [38]. An enhanced expression of CCL17/TARC has been reported in several human malignancies, such as Hodgkin’s and B cell lymphoma [39, 40]. Previous studies have revealed that CCL17/TARC possesses several important effects attributed to tumor growth, such as the proliferation [41-43], migration and recruitment of regulatory T-cells [44-46].

We detected a higher expression of CCL17/TARC in MCPyV-positive MCC compared to MCPyV-negative MCC cells at both the RNA and protein level. Furthermore, by co-transfecting MCC cells with MCPyV full LT, MKL-1 LT, MKL-2 LT and MS-1 LT expression plasmids, as well as CCL17/TARC promoter, resulted in an upregulation of CCL17/TARC promoter activity (Figure 2), which in turn increased the expression of CCL17/TARC (Figure 4). At present, we do not know why the different LT variants activate the CCL17 promoter at different levels. MCPyV full-length LT can interact with several transcription factors including Brd4, the E2F family members 2 and 3, MED14/CRSP2, PRTF1, SALL2 and DP1 (see supplementary data in Moens et al. [47]). However, no putative binding sites are present in the CCL17 promoter fragments used in our study. The truncated MCPyV LT variants MKL-1, MKL-2 and MS-1 retain the pRb binding motif and may thus relieve the pRb-mediated inhibition of E2F by using pRb. The CCL17/TARC promoter contains the putative GGCGGCA E2F binding site at position -1085/-1079 [48]. This means that our luciferase reporter plasmids containing the CCL17/TARC promoter fragments -2535/+40 and-1084/+40 contain this putative E2F binding site. LT may thus activate the CCL17/TARC promoter fragments with an E2F motif by usurping pRb and releasing the repressive effect on the E2F site. However, the shorter promoter fragment (-375/+40) which lacks the putative E2F binding site is still trans-activated by LT, indicating that LT-mediated activation seems to be independently of the E2F site. The autocrine release of CCL17/TARC results in an increase of MCPyV, both in early and late promoter activity, as shown by the overexpression of CCL17 with pCMV2-CCL17-flag plasmid and stimulation with recombinant CCL17/TARC (Figure 3). These data above indicated that MCPyV LT increases CCL17/TARC expression, which results in the replication of MCPyV and the release of viral oncoproteins, which in turn promotes MCC development. In line with our data, the virus-mediated expression of CCL17/TARC has been reported in different types of cells. The Epstein-Barr infection of B cells induces the expression of CCL17/TARC [49], whereas the respiratory syncytial virus infection of Balb/c mice results in an increased CCL17/TARC production in the lung [50]. Hence, the virulent properties of viruses may depend on their ability to stimulate the expression of CCL17/TARC.

The role of CCR4 in tumor growth and survival has previously been reported. CCR4 is expressed in T-cell leukemia [51], non-lymphoid solid tumors, such as breast cancer, lung cancer, colorectal cancer, gastric and hepatocellular carcinoma [42, 43, 52-56], where it may contribute to the proliferation of tumor cells and chemotaxis of regulatory T cells [43, 46, 53, 57]. Interestingly, an elevated expression of CCR4 in different types of human cancers has been related to a poor prognosis [52, 58-61].

By immunohistochemistry, we detected CCL17/TARC and its receptor CCR4 in the tumor cells of all MCC tissue samples analyzed. The normal epidermis of the skin was also shown to express CCL17/TARC and CCR4, and has been reported earlier [62-65]. We did not observed any difference in the protein expression of CCR4 and CCL17/TARC between MCPyV-negative and MCPyV-positive primary cutaneous MCCs. Since immunohistochemistry is more qualitative rather than a quantitative analysis therefore, increase in CCL17/TARC levels between MCPyV-positive and -negative samples may not be visible by IHC.

Given the fact that MCC produces CCL17/TARC and expresses CCR4, we decided to investigate the effect of exogenously added CCL17/TARC on intracellular signaling pathways. Recombinant hCCL17/TARC induced the proliferation in MCPyV-negative MCC cells, which was abolished in the presence of the CCR4 receptor antagonist. The addition of CCL17/TARC resulted in ERK1/2 phosphorylation in MCC13 cells (Figure 6). Previous studies have shown that CCL17/TARC induced chemotaxis of the mouse T-cell lymphoma cell line EL4 in a MEK1/2-ERK1/2-dependent manner [66].

We also demonstrate that CCL17/TARC activates the NF-κB pathway in MCC13 cells. Previous studies have shown that CCL17/TARC is a NF-κB target gene [67, 68], but it has not been shown that CCL17/TARC can itself activate NF-κB. CCR4-mediated MMP13 activity in colorectal cancer cells requires NF-κB [52]. Chemokine-like factor (CKLF1), which also uses the CCR4 receptor, can activate the NF-κB pathway [69], and NF-κB signaling is significantly downregulated in CCR4^-/-^ macrophages [70].

In the tumor microenvironment, a high expression of CCL17/TARC and CCR4 by MCC cells may contribute to the activation of inflammatory pathways and the promotion of tumor growth and immune suppression. We found that CCL17/TARC stimulated proliferation of MCC13 cells, whereas several studies have reported that CCL17/TARC or CCL22-associated CD4^+^CD25^+^Foxp3^+^ increases the population in tumor-infiltrating lymphocytes (TILs), with peripheral blood lymphocytes (PBLs) being one of the reasons for impaired anti-tumor immunity in both gastric and esophageal squamous cell carcinoma [44, 45]. It is postulated that the preferential attraction of CCR4-bearing Th2 lymphocytes may cause a shift towards a Th2-dominated cytokine microenvironment, thereby hampering the cytotoxic immune response and providing a mechanism by which neoplastic cells are able to escape from the immune system [71]. Targeting CCR4 is an emerging strategy for immunotherapy for cancer [57, 72]. Gain-of-function mutations in adult T cell lymphoma have been reported [73] and recently, Mogalizumab, a monoclonal antibody targeting CCR4 receptor has shown promising results in the treatment of relapsed adult T cell lymphoma patients [74-78].

Taken together, we demonstrate that MCPyV LT is linked with an altered expression of CCL17/CCR4. The expression of CCL17/TARC and CCR4 may constitute an autocrine or paracrine survival loop which contributes to the growth and survival of the tumor, and also mediates immune suppression through the recruitment of regulatory T cells. Thus, strategies based on the selective targeting of the CCL17/CCR4 axis, either by monoclonal antibodies or specific receptor antagonists, could be a therapeutic interventions for patients with MCC.

## MATERIALS AND METHODS

### Materials

In our study, the following primary antibodies were used: i.e. monoclonal mouse MCPyV LTA (Sc-136172, Santa Cruz Biotechnologies), polyclonal rabbit CCL17/TARC (Ab-182793, Abcam), polyclonal rabbit anti-CCR4 (cat.#PA1516, Boster, USA), polyclonal rabbit ERK2 (Sc-136172, Santa Cruz Biotechnologies, Dallas, TX, USA), monoclonal rabbit keratin 20 (cat.# 13063, Cell Signaling, Danvers, MA, USA), monoclonal rabbit Phospho-p44/42 MAPK (Erk1/2) (Thr202/Tyr204) (cat.#4370 Cell Signaling), monoclonal rabbit p44/42 MAPK (Erk1/2) (cat.#4695S, Cell Signaling), Phospho-NF-κB p105 (Ser933) (18E6) (cat.#4806S, Cell Signaling) rabbit anti-GAPDH (cat.# G9545, Sigma Aldrich, St. Louis, MO, USA) and CCR4 chemokine receptor antagonist (C 021 dihydrochloride) (cat. #3581, Tocris Bioscience Minneapolis, MN, USA)).

### Plasmids

The empty expression plasmid pcDNA3.1(+) was purchased from Invitrogen. pcDNA3-full large T-antigen (pcDNA6.MCV.cLT206.V5_CM2B4) was purchased from Addgene (Cambridge, MA, USA), while pcDNA3-MKL-1 large T-antigen, pcDNA3-MKL-2 large T-antigen and pcDNA3-MS-1 large T-antigen were constructed by site-directed mutagenesis using the original pcDNA6.MCV.cLT206.V5_CM2B4 plasmid. pCMV2-CCL17-flag (cat.# HG10233-M-F) expression plasmid was purchased from SinoBiological (Beijing, China). The plasmids pCCL17-2532+40-LUC, pCCL17-1080/+40-LUC, and pCCL17-375/+40-LUC containing CCL17 promoter fragments in the luciferase reporter plasmid pGL3-basic (Promega) were a kind gift from Dr. Daniel Hebenstreit [79].

### Cell lines and human tissue samples

MCC13 and MKL-2 cell lines were kindly provided by Dr. Baki Akgül (University of Cologne, Germany). MCC13 is a MCPyV-negative MCC cell line, whereas MKL-2 is a MCPyV-positive MCC cell line [58, 59]. MCC13 cells were grown in RPMI-1640 with 10% FBS in the presence of 100 μg/ml streptomycin and 100 units/ml of penicillin, while MKL-2 cells were grown in RPMI-1640 with 20% FBS in the presence of 100 μg/ml streptomycin and 100 units/ml penicillin. Cells were kept in a humidified CO_2_ incubator at 37°C. Human MCC tissue were obtained during 2000-2015 from the St. Olavs University Hospital Trondheim, Norway according to the ethical approval from the Regional Ethical Committee (REK NORD application number 2016/988).

### PCR-based site-directed mutagenesis of MCPyV truncated LT encoding plasmid

To generate expression plasmids encoding truncated variants of MCPyV LT expressed in the virus-positive MKL-1, MKL-2 and MS-1 MCC cell lines, an oligonucleotide-directed mutagenesis was performed using the QuickChange site-directed mutagenesis kit from Stratagene (cat. no. 200518; Stratagene La Jolla, CA, USA). Plasmid pcDNA6.MCV.cLT206.V5 was used as a template to generate the plasmids pcDNA3-MKL-1 LT, pcDNA3-MKL-2 LT, pcDNA3-MS-1 LT. Table 1 shows different primers to generate truncated MKL-1, MKL-2 and MS-1 sequences from full-length LT.

**Table 1:** Primer sequences for full-length LT and generating truncated Large Tag transcripts

MCPyV full-length LT sequence primers	
Full_LT.F	5′-TACAAGCACTCCACCAAAGC-3′
Full_LT.R	5′-TCCAATTACAGCTGGCCTCT-3′
Site-directed mutagenesis primers to make MKL-1 LT	
MKL-1_LTstop.F	5′-GCCATGCTGTGTACAAGTTTTAAACAGTCTCCTGTTTTGC-3′
MKL-1_LTstop.R	5′-GCAAAACAGGAGACTGTTTAAAACTTGTACACAGCATGGC-3′
Site-directed mutagenesis primers to make MKL-2 LT	
MKL-2_LTstop.F	5′-GAAGACCCCTCCTCCATAGTCAAGAAAGCG-3′
MKL-2_LTstop.R	5′-CGCTTTCTTGACTATGGAGGAGGGGTCTTC-3′
Site-directed mutagenesis primers to make MS-1 LT	
MS-1_LTstop.F	5′-GCCACTGCTAAATTAGGAATTTCAAGAAAAAG-3′
MS-1_LTstop.R	5′-CTTTTTCTTGAAATTCCTAATTTAGCAGTGGC-3′

### Transfection

Cells were seeded out in 6- and 12-well cell culture plates with a total number of 1.5x10^5^ and 2x10^5^, respectively. At the time of transfections, the cells were approximately 60-70% confluent. jetPRIME (Polyplus-transfection SA, Illkirch, France) was used to transfect all plasmids according to the manufacturer’s instructions. A total of 2-μg per well in a 6-well plate and 800 ng per well in a 12-well plate DNA was used to transfect cells. All experiments were performed 24 hrs after transfection.

### RNA extraction

RNA extraction was done by using RNeasy® Plus Mini kit (Qiagen, Hilden, Germany) according to the manufacturer’s protocol. For RNA yield and quality, A_260/A280_ and A_260/A230_ ratios were analyzed with Nano-Drop® ND-2000 spectrophotometer (NanoDrop Technologies, ThermoFisher Scientific, Waltham, MA, USA).

### cDNA construction and quality control

iScript™ cDNA Synthesis Kit (BioRad, Hercules, CA, USA) was used to make cDNA. A total of 1-2 μg RNA was used to generate cDNA according to the manufacturer‘s instructions. PCR with the housekeeping APRT primers (5’- CCCGAGGCTTCCTCTTTGGC-3’ and 5’-CTCCCTGCCCTTAAGCGAGG-3’) were used to check for genomic DNA contamination in the cDNA prep. An 800 bp fragment was obtained with genomic DNA as a template, while a 300 bp amplicon was obtained with cDNA (76). PCR products were visualized on a 1% agarose gel stained with Gelred™ Nucleic Acid Gel Stain gel red stain (Biotium, Cambridge Bioscience, Bar Hill, UK).

### RT^2^ Profiler PCR array

A human cytokines and receptor genes transcription was measured using the human RT^2^ Profiler PCR Inflammatory Cytokines and Receptors Array (PAHS-011ZA, SABiosciences, Qiagen). Twenty μl cDNA were diluted to 111 μl by adding 91 μl of RNase-free water. One hundred and two μl were added in a 1350 μl 2x RT^2^ SYBR Green Mastermix according to the manufacturer’s protocol. Twenty-five μl PCR components mix was added to each well of a 96-well plate. A two-step real-time PCR was initiated at 95°C (10 min) for one cycle, and followed by alteration of 95°C (15 sec) and 60°C (1 min) for 45 cycles by using Light Cycler 96 (Roche Diagnostics, Indianapolis, IN, USA). All data was collected from the PCR machine by Light Cycler 96 SW 1.1 software (Provided by manufacturer), and analyzed by SA Bioscience’s Gene Glob PCR Array Data Analysis Web Portal. For considering a gene differentially expressed, we used a differential cut-off of 2-fold (up- or downregulated).

### Luciferase assays

For luciferase assays, approximately 24 hrs after transfection, cells were lysed in a 100 μl Luciferase Assay Tropix Lysis solution (ThermoFisher Scientific), with 0.5 mM DTT. Cells were scraped, transferred to Eppendorf tubes and then centrifuged for 3 minutes at 12,000 g. Twenty μl of the supernatant was used in a 96-well microtiter plate, and a 50 μl luciferase buffer (Promega, Madison, WI, USA) was added. A Luminometer (Labsystem, Luminoscan RT) used to measure lights units. Each experiment was repeated three times with three independent parallels for each experiment, and luciferase values were corrected for protein content in each sample. The total protein concentration was measured using the MN protein quantification assay (Macherey-Nagel GmbH, Düren, Germany).

### Quantitative real-time PCR

The gene expression level of CCL17/TARC was measured by real-time quantitative RT-PCR using an ABI PRISM^®^ 7300 Sequence Detection System (Applied Biosystems, Foster City, CA, USA). The expression level was measured by using a FAM-labeled TaqMan gene expression assay CCL17/TARC probe/primer (Cat. # Hs00171074_m1), and the expression level was normalized by using a VIC/MGB probe/primer Eukaryotic 18S rRNA Endogenous Control (Cat. #4319413E, Applied Biosystems). PCR reactions were prepared in a total volume of 25 μl, with a final concentration of 1X TaqMan^®^ Universal Master Mix and cDNA from 1 μg total RNA.

### Immunoblotting

Western blot was done by running samples in 4-12% of NuPAGE Bis-Tris Mini Gels (Invitrogen Life Technologies, Carlsbad, CA, USA) according to the manufacturer’s protocol and blotted onto a 0.45 μm PVDF membrane (Millipore, Billerica, MA, USA). Membrane blocking was performed by using TBS-T (TBS with 0.1% Tween-20; Sigma Aldrich) containing 5% (w/v) dried skimmed milk for 1 hour. The protein was probed by using an appropriate primary antibody overnight at 4°C. After washing the membrane 3 times with TBS-T, an appropriate secondary antibody was added for 1 hour at room temperature. After 2 washes with TBS-T and 2 washings with washing buffer, antigen-antibody complex was visualized by using SuperSignal™ West Pico Chemiluminescent Substrate (Cat.#34080 Thermo Fisher Scientific, Rockford, IL, USA). Magic-Mark™ Western standard from Invitrogen Life Technologies was used to estimate the molecular mass of the detected proteins.

### Immunohistochemistry

Formalin-fixed and paraffin-embedded tissue sections were deparaffinized in xylene and graded alcohols, hydrated and washed in PBS. After antigen retrieval in a sodium citrate buffer (pH 6) in a microwave oven, the endogenous peroxidase was blocked by 0.3% H_2_O_2_ for 15 min. Sections were incubated overnight at 4°C with the primary antibody CCR4 (Abcam Cat.#ab1699, Cambridge, UK), CCL17/TARC (Abcam Cat.#ab182793), MCPyV-LT (Santa Cruz Biotechnology Cat.#sc-136172) and CK20 (Roche Cat.#790-4431). As a secondary antibody, the anti-rabbit-HRP SuperPicTure Polymer detection kit (87-9663, Zymed-Invitrogen, San Francisco, CA, USA) or anti-mouse EnVision-HRP (Dako, Agilent Technologies, Inc., Santa Clara, CA, USA) was used. A matched isotype control was used as a control for nonspecific background staining.

### MTT assay

To measure cell proliferation, the colorimetric MTT (3-(4,5-dimethylthiazol-2-yl)-2,5-diphenyltetrazodium bromide)-assay was used [80].

### Statistical analysis

The RT2 Profiler PCR Array data analysis version 3.5 (http://dataanalysis.sabiosciences.com/pcr/arrayanalysis.php) was used for inflammatory cytokines and receptor data analysis. For the analysis, the significant values were considered with a fold change/fold regulation ≥2 and a p-value less than 0.05. GraphPad software was used for the statistical analysis and graphs. The sample *t*-test was used to compare differences between the experimental and control group and a p-value set <0.05. The densitometry analysis of Western blot was done by using imageJ.

## SUPPLEMENTARY MATERIALS FIGURES


