# Supplementary material for: CCL17/TARC and CCR4 expression in Merkel cell carcinoma
Source: Oncotarget. 2018 Jul 31;9(59):31432–47. doi: 10.18632/oncotarget.25836 (PMC6101144; doi:10.18632/oncotarget.25836)
Supplement: Supplementary file 1 [file oncotarget-09-31432-s001.pdf]

CCL17/TARC and CCR4 expression in Merkel cell carcinoma

SUPPLEMENTARY MATERIALS

```
>gi|531990549|dbj|BAN78688.1| large T antigen [Merkel cell polyomavirus]
MDLVLNRKEREALCKLLEIAPNCYGNIPLMKAAFKRSCLKHHPDKGGNPVIMMELNTLWSKF
QQNIHKLRSDFSMFDEVDEAPIYGTTKFKEWWRSGGFSFGKAYEYGPNPHGTNSRSRKPSSN
ASRGAPSGSSPPHSQSSSSGYGSFSASQTSDSQSRGPDIPPEHHEEPTSSSGSSSREETTNS
GRESSTPNGTSVPRNSSRTDGTWEDLFCDESLSSPEPPSSSEEPEEPPSSRSSPRQPPSSSA
EEASSSQFTDEECRSSSFTTPKTPPPFSRKRKFGGSRSSASSASSASFTSTPPKPKKNRETP
VPTDFPIDLSDYLSHAVYSNKTVSCFAIYTTSDKAIELYDKIEKFKVDFKSRHACELGCILL
FITLSKHRVSAIKNFCSTFCTISFLICKGVNKMPEMYNNLCKPPYKLLQENKPLLNYEFQEK
EKEASCNWNLVAEFACEYELDDHFIILAHYLDFAKPFPCQKCENRSRLKPHKAHEAHHSNAK
LFYESKSQKTICQQAADTVLAKRRLEMLEMTRTEMLCKKFKKHLERLRDLDTIDLLYYMGGV
AWYCCLFEEFEKKLQKIIQLLTENIPKHRNIWFKGPINSGKTSFAAALIDLLEGKALNINCP
SDKLPFELGCALDKFMVVFEDVKGQNSLNKDLQPGQGINNLDNLRDHLDGAVAVSLEKKHVN
KKHQIFPPCIVTANDYFIPKTLIARFSYTLHFSPKANLRDSLDQNMEIRKRRILQSGTTLLL
CLIWCLPDTTFKPCLQEEIKNWKQILQSEISYGKFCQMIENVEAGQDPLLNILVEEEGPEET
EETQDSGTFSQ
```

Supplementary Figure 1: Amino acids sequence of full-length MCPyV LT. The amino acids marked in red represent the last amino acid of the truncated LT in MS-1 (N), MKL-1 (Y) and MKL-2 (F). Green amino acid are residues that differ in MKL-2 LT compared to MKL-1 and MS-1 LT.

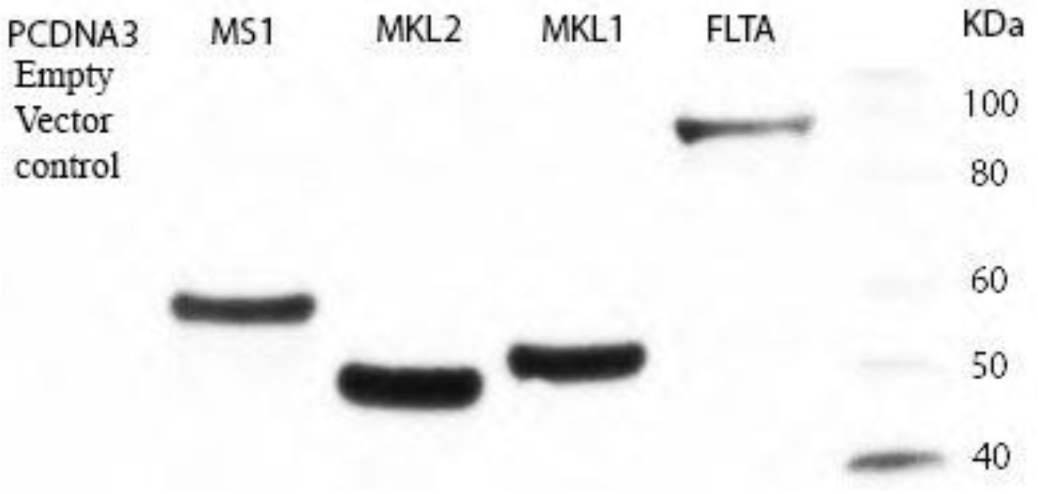

Supplementary Figure 2: Western blot detection of MCPyV full-length and truncated (MKL-1, MKL-2 and MS-1) LT in MCC13 cells. Cells were transfected with an empty control vector (pcDNA3) or expression plasmid for full-length (FLTA) and truncated (MKL-1, MKL-2 and MS-1) LT. Lysate from full-length LT showed a band at approximately 100KDa, and lysates from cells transfected with an expression plasmid for truncated MKL-1, MKL-2 and MS-1 LT showed a band at approximately 50KDa, 45KDa and 60KDa, respectively. The molecular mass marker is shown in the utmost right lane.
